# Racial Disparities in Shared Decision-Making and the Use of mHealth Technology Among Adults With Hypertension in the 2017-2020 Health Information National Trends Survey: Cross-Sectional Study in the United States

**DOI:** 10.2196/47566

**Published:** 2023-09-13

**Authors:** Yuling Chen, Suratsawadee Kruahong, Sabrina Elias, Ruth-Alma Turkson-Ocran, Yvonne Commodore-Mensah, Binu Koirala, Cheryl R Dennison Himmelfarb

**Affiliations:** 1 Johns Hopkins School of Nursing Baltimore, MD United States; 2 Mahidol University Faculty of Nursing Bangkok Thailand; 3 Beth Israel Deaconess Medical Center Harvard Medical School Boston, MA United States; 4 Johns Hopkins Bloomberg School of Public Health Baltimore, MD United States; 5 Johns Hopkins School of Medicine Baltimore, MD United States

**Keywords:** mobile health, disparities, shared decision-making, hypertension, association, decision-making, mHealth, technology, health disparity, adult, smartphone, racial, ethnic, health literacy, digital literacy

## Abstract

**Background:**

Mobile health (mHealth) technology has the potential to support shared decision-making (SDM) and improve hypertension control. However, our understanding of the variations in individuals’ involvement in SDM and mHealth usage across different racial and ethnic groups in the United States is still limited.

**Objective:**

This study aimed to investigate the extent of involvement in SDM and the usage of mHealth technology in health-related activities among US adults with hypertension from diverse racial and ethnic backgrounds and to examine whether the mHealth usage differed by individuals’ level of engagement in SDM.

**Methods:**

This study used cross-sectional data from the 2017 to 2020 Health Information National Trends Survey, which was conducted on US adults with self-reported hypertension, and race and ethnicity data were included. The exposure of interest was race and ethnicity. The outcomes were SDM and mHealth usage. SDM was assessed using an item: “In the past 12 months, how often did your health professional: involve you in decisions about your healthcare as much as you wanted?” mHealth usage was defined as using a smartphone or tablet to engage in (1) making health decisions, (2) discussing health decisions with health providers, (3) tracking health progress, and (4) sharing health information. Weighted multivariable logistic regression models were used to examine the association between race and ethnicity and SDM or mHealth usage adjusted for covariates and stratified by the level of engagement in SDM.

**Results:**

This study included 4893 adults with hypertension, and the mean age was 61 (SD 13) years. The sample was 53% female, 61% (n=3006) non-Hispanic White, 19% (n=907) non-Hispanic Black or African American, 12% (n=605) Hispanic, 4% (n=193) non-Hispanic Asian, and 4% (n=182) non-Hispanic other. Compared to the non-Hispanic White adults, non-Hispanic Black adults were more likely to use mHealth to make health decisions (adjusted odds ratio [aOR] 1.70, 95% CI 1.23-2.34), share health information (aOR 1.46, 95% CI 1.02-2.08), and discuss health decisions with health providers (aOR 1.38, 95% CI 1.02-1.87). Significant associations were observed specifically among those who were always involved in SDM. Asian adults were less likely to be involved in SDM (aOR 0.51, 95% CI 0.26-0.99) and were more likely to use mHealth to track progress on a health-related goal (aOR 2.07, 95% CI 1.28-3.34) than non-Hispanic White adults. Hispanic adults were less likely to use mHealth to share health information (aOR 0.47, 95% CI 0.33-0.67) and discuss health decisions with health providers (aOR 0.65, 95% CI 0.46-0.94) compared to non-Hispanic White adults.

**Conclusions:**

This study observed racial and ethnic disparities in SDM and mHealth usage among US adults with hypertension. These findings emphasize the significance of comprehending the involvement of SDM and the usage of mHealth technology within racially and ethnically diverse populations.

## Introduction

Hypertension is the leading preventable risk factor for cardiovascular disease and premature death globally [1]. Population-level hypertension management is a global public health priority [1]. Almost half (47%) of adults in the United States have hypertension, and of those, less than half (43.7%) had controlled hypertension in 2017-2018 [2,3]. Despite progress made in improving hypertension control rates in the 1990s and early 2000s, the prevalence of uncontrolled hypertension worsened in recent years [2,4]. In addition, substantial racial and ethnic disparities in the prevalence, awareness, treatment, and control of hypertension persist [2,5]. Particularly, non-Hispanic Black, non-Hispanic Asian, and Hispanic adults have worse rates of uncontrolled hypertension when compared with non-Hispanic White adults [2,5].

Hypertension control is influenced by multiple determinants [3], and the causes of racial and ethnic disparities in hypertension control are multifaceted and encompass various factors [6]. One key factor among them is the quality of physician-patient interaction and communication [7,8]. Enhancing physician-patient interaction can be achieved through the implementation of shared decision-making (SDM), a communication process by which patients and clinicians collaborate to choose tests, treatments, and care plans that most align with available evidence and individual patients’ preferences and values [9-12]. The latest national guidelines for hypertension prevention have emphasized the importance of promoting SDM to enhance hypertension control and address existing disparities in hypertension management [13-15]. Evidence suggests that SDM can result in more appropriate care, less overtreatment, better health outcomes, and lower health care treatment costs [16,17]. Despite widespread calls for SDM to be embedded in health care, there is limited evidence to inform approaches for SDM in hypertension care, with few studies demonstrating the benefits of SDM interventions for hypertension control [17-19]. While prior research has provided evidence of racial and ethnic disparities in patients’ treatment preferences for hypertension management [10] and SDM among individuals receiving usual care in the United States [20], the presence of racial and ethnic disparities in the engagement of SDM specifically among adults with hypertension in the United States remains uncertain and requires further investigation.

Mobile health (mHealth) technology, which involves the use of mobile and wireless technologies such as smartphones and tablets, has rapidly advanced in its role of supporting the management of chronic diseases, including hypertension [21]. There is evidence indicating that mHealth has demonstrated promise in improving self-management of health behaviors [22], enhancing patient-provider interaction [23], and improving hypertension control [21,24]. Moreover, some studies have demonstrated that the integration of mHealth technology can significantly enhance the opportunities for SDM and foster increased patient engagement in SDM processes [25,26].

While mHealth technology holds significant potential benefits for health care, it is important to acknowledge the existence of digital divides in the general population in the United States, which is characterized by disparities in race and ethnicity as well as other factors such as income, educational attainment, and health literacy [27]. Several studies have explored racial and ethnic disparities in mHealth usage, but much of the research has primarily focused on specific technologies (eg, patient portals) [28] or general technology use among older adults [29]. While some studies indicate the potential of mHealth technology to reduce disparities [21,30], conflicting evidence indicates that if only individuals with greater resources have access to these technologies, it may inadvertently widen racial disparities [31]. However, our understanding of the variations in mHealth usage across different racial and ethnic groups in the United States is still limited [28]. Moreover, there is a lack of understanding regarding how the usage of mHealth for specific health care activities, such as making healthy decisions and sharing health information with health care providers, differs across subgroups of individuals with hypertension, specifically in terms of race and ethnicity. In addition, while mHealth technology has been proposed to enhance SDM [32], it is currently unclear whether the usage of mHealth varies among different racial and ethnic groups based on their level of involvement in SDM.

Therefore, our study aims to investigate the extent of involvement in SDM and the usage of mHealth technology in health-related activities among US adults with hypertension from diverse racial and ethnic backgrounds, using cross-sectional data from the 2017 to 2020 Health Information National Trends Survey (HINTS). Additionally, the study aims to examine whether the usage of mHealth technology in health-related activities among adults with hypertension differed by their level of engagement in SDM.

## Methods

### Study Design and Setting

This analysis used data from the HINTS, a nationally representative mailed survey administered in the United States by the National Cancer Institute of noninstitutionalized US adults aged ≥18 years. HINTS is a cross-sectional study that collects data regularly about the American public’s knowledge of, attitudes toward, and use of cancer-related information and various aspects of digital health, including mHealth use and access. Since 2003, HINTS has been administered every few years. There are several versions of HINTS administration (eg, HINTS 1, HINTS 2, HINTS 4, and HINTS 5), and each version includes several cycles. The full description of the HINTS methodologies can be found elsewhere [33]. In this study, we pooled the HINTS 5 version, cycles 1-5 data sets administered from 2017 to 2020 to increase the precision of estimates for minority subpopulations. The overall household response rate from the 2017 to 2020 mailed survey ranged from 30.2% to 36.7% [34].

### Sampling and Stratification

In HINTS 5 cycles 1-5, the sampling strategy followed a 2-stage design [33]. Initially, a stratified sample of addresses was selected from a database of residential addresses. In the subsequent stage, 1 adult was chosen from each sampled household. The sampling frame of addresses was divided into 2 explicit sampling strata based on the concentration of minority populations. The purpose of establishing these high- and low-minority strata was to enhance the accuracy of estimates for minority subpopulations by oversampling the high-minority stratum. This oversampling technique aimed to increase the sample size for minority subpopulations. By incorporating an oversampling strategy in the high-minority stratum, the study sought to provide a more extensive representation of individuals from areas with a high concentration of minority populations, resulting in more robust statistical analysis and more precise estimates for these subpopulations.

### Study Population

Individuals with data available on self-reported hypertension, race and ethnicity, and any mHealth usage information were included in this analysis. Individuals with self-reported hypertension were determined by a single question: “Has a doctor or other health professional ever told you that you had high BP or hypertension?” The response options were yes or no. Individuals responding “yes” were ascertained as having high BP or hypertension. After excluding adults without hypertension (n=8959), those who had missing data on race or ethnicity (n=888), and any mHealth usage (see definition in “Mobile Health Usage” section below, n=1352), the final analysis included 4893 adults with hypertension.

### Race or Ethnicity

The exposure of interest of the study is race or ethnicity. This variable was derived from the combination of 2 self-reported variables: race and ethnicity. Race was determined through a single-item question asking participants about their race using the US Census definitions, with 14 possible responses including options such as White, Black or African American, American Indian or Alaska Native, Asian Indian, and others. The ethnicity variable was determined through a single-item question asking participants if they are of Hispanic, Latino, or Spanish origin, with response options including “no” or “yes.” The combination methodology of the race and ethnicity variables is outlined in the HINTS 5 History Document [35]. In relation to the aforementioned items pertaining to race and ethnicity, the HINTS data set offers a variable named “race/ethnicity” that encompasses several categories, including non-Hispanic White, non-Hispanic Black or African American (non-Hispanic Black), Hispanic, non-Hispanic Asian, and non-Hispanic other.

### Shared Decision-Making

We used a single HINTS item to assess the level of participant involvement in SDM based on previous studies [36,37]. This particular HINTS item was used in the Health Communication and Health Information Technology (HC/HIT) objective (HC/HIT-3) of Healthy People 2020—“increase the proportion of persons who report that their health care providers ‘always’ involved them in decisions about their health care as much as they wanted” [37]. The baseline data for the Healthy People 2020 national goals and objectives were established using this specific HINTS question, which was also used to assess SDM in our study [37]. The single item is “In the past 12 months, how often did your health professional: involve you in decisions about your healthcare as much as you wanted?” A 4-point Likert scale was used to assess the SDM, which includes “always,” “usually,” “sometimes,” and “never.” We dichotomized the variable as “always involved in SDM” and “usually/sometimes/never involved in SDM” following the Healthy People 2020 objective (HC/HIT-3) and previous studies [36-38].

### mHealth Usage

The use of mHealth technology in this study is defined as using the smartphone or tablet to engage in the following health care activities: (1) making health decisions—“Has your tablet or smartphone helped you make a decision about how to treat an illness or condition?” (2) discussing health decisions with health providers—“Has your tablet or smartphone helped you in discussions of health decisions with your health providers?” (3) tracking health progress—“Has your tablet or smartphone helped you track progress toward a health-related goal, such as quitting smoking, losing weight, or increasing physical activity?” and (4) sharing health information—“Have you shared health information from either an electronic monitoring device or smartphone with a healthcare professional within the last 12 months?” The responses to the above 4 questions were “yes” or “no.” In this study, any mHealth usage was defined as mHealth use in any of the 4 health-related activities described above.

### Covariates

Covariates examined included age, gender, educational level, household income, marital status, health insurance, location (urban or rural), BMI, current smoking status (including cigarettes and e-cigarette use), depression, chronic diseases including self-reported heart condition (eg, heart attack, angina, or congestive heart failure), diabetes, lung disease, and cancer [39,40]. All covariates were assessed at the time of the survey.

### Statistical Analyses

We described the demographic characteristics and mHealth usage using unweighted and weighted percentages by race or ethnicity. We used the survey weighting and Taylor series variance estimation to calculate the prevalence estimated and SEs [33]. We performed survey-weighted Pearson chi-square tests to compare the demographic variables and mHealth usage by race or ethnicity.

To examine the association between race or ethnicity and SDM or mHealth usage, we performed unweighted and weighted multivariable logistic regression models with survey weighting. To determine whether the association between race or ethnicity and mHealth usage differs by SDM, we performed multivariable logistic regression models stratifying by SDM (always involved in SDM vs usually/always/never involved in SDM). To determine whether mHealth usage is associated with SDM, we performed unweighted and weighted multivariable logistic regression models with survey-weighting, using mHealth usage as the independent variable and SDM as a dependent variable. All logistic regression models were adjusted for age, gender, educational levels, household income, marital status, location, health insurance, BMI, current smoking status, depression, and chronic diseases including self-reported heart condition, diabetes, lung disease, and cancer.

Statistical analyses were performed using Stata/SE 17.0 (Stata Corp LLC). Adjusted odds ratio (aOR) and 95% CI were calculated for multivariable logistic regression models. A 2-sided *P* value of <.05 was considered statistically significant for all analyses.

### Ethical Considerations

HINTS was approved by the Westat Institutional Review Board. It was classified as exempt by the US National Institutes of Health Office of Human Subjects Research Protections due to the deidentification of the data.

## Results

### Sample Characteristics

The final analysis included 4893 adults with hypertension, and the mean age was 61 (SD 13) years. The sample was 53% female, 61% (n=3006) non-Hispanic White, 19% (n=907) non-Hispanic Black, 12% (n=605) Hispanic, 4% (n=193) non-Hispanic Asian, and 4% (n=182) other non-Hispanic adults. There were significant differences in age, sex, education, household income, marital status, insurance, BMI, current smoking status, depression, history of diabetes, lung disease, and cancer among the different race or ethnicity groups (Table 1). Table S1 in Multimedia Appendix 1 presents the unweighted percentages of demographic characteristics and clinical data.

**Table 1:** Weighted percentage of demographic characteristics and clinical data among adults with hypertension (N=4893).

Characteristics		All, %	Non-Hispanic White, %	Non-Hispanic Black, %	Hispanic, %	Non-Hispanic Asian, %	Non-Hispanic other, %	*P* value	
**Age (years)**									<.001
	18-34	9	8.3	10.5	6.1	19.3	17.8		
	35-49	25.4	22.5	27.1	40.7	26.2	20.5		
	50-64	39.4	39.2	42.7	36.6	30.7	51.1		
	65-74	16.6	18.5	15.0	10.6	13.9	8.0		
	75+	9.6	11.6	4.7	5.9	9.9	2.6		
**Sex**									<.001
	Female	45.9	43.4	63.4	37.0	37.5	61.0		
	Male	54.1	56.6	36.6	63.0	62.5	39.0		
**Education**									<.001
	Less than high school	7.8	4.7	10.0	20.0	15.9	9.4		
	High school graduate	22.8	22.0	29.3	23.3	6.3	26.3		
	Some college	41.3	44.6	34.9	36.3	17.9	46.9		
	Bachelor degree	28.0	28.7	25.7	20.4	59.9	17.4		
**Household income (US $)**									<.001
	<$20,000	15.3	12.7	25.9	18.1	7.1	18.3		
	$20,000-$35,000	11.2	9.6	18.2	14.1	8.3	5.1		
	$35,000-$50,000	14.7	12.3	16.3	23.3	11.4	29.8		
	$50,000-$75,000	20.4	21.1	17.8	15.4	35.0	19.5		
	≥$75,000	38.4	44.2	21.8	29.1	38.3	27.2		
**Marital status**									<.001
	Married	41.0	36.9	60.4	41.5	34.0	46.9		
	Others^a^	59.0	63.1	39.6	58.5	66.0	53.1		
**Insurance**									.004
	No	5.5	3.9	9.7	10.3	1.3	6.7		
	Yes	94.5	96.1	90.3	89.7	98.7	93.3		
**Location**									.007
	Urban	15.1	18.2	8.2	6.8	7.5	21.7		
	Rural	84.9	81.8	91.8	93.2	92.5	78.3		
**BMI**									.07
	<25 kg/m^2^	17.1	18.2	12.3	14.8	28.5	12.2		
	≥25 kg/m^2^	82.9	81.8	87.7	85.2	71.5	87.8		
**Current smoking**									<.001
	No	83.4	81.6	89.7	88.4	89.3	67.7		
	Yes	16.6	18.4	10.3	11.6	10.7	32.3		
**Depression**									.01
	No	69.9	69.8	73.4	67.3	85.2	49.5		
	Yes	30.1	30.2	26.6	32.7	14.8	50.5		
**Heart condition**									.40
	No	85.0	84.7	88.1	86.1	80.0	77.6		
	Yes	15.0	15.3	11.9	13.9	20.0	22.4		
**Diabetes**									<.001
	No	66.9	69.9	64.1	51.1	62.1	76.8		
	Yes	33.1	30.1	35.9	48.9	37.9	23.2		
**Lung disease**									.06
	No	84.8	86.0	85.5	78.5	87.5	74.9		
	Yes	15.2	14.0	14.5	21.5	12.5	25.1		
**Cancer**									<.001
	No	88.7	86.5	91.2	94.2	96.4	95.6		
	Yes	11.3	13.5	8.8	5.8	3.6	4.4		
**SDM^b^**									.06
	Usually/sometimes/never	43.5	43.5	35.1	48.2	59.9	48.4		
	Always	56.5	56.5	64.9	51.8	40.1	51.6		

^a^Including divorced, widowed, separated, living as married, and single.

^b^SDM: shared decision-making.

### Association Between Race or Ethnicity and mHealth Usage

**Figure 1:**
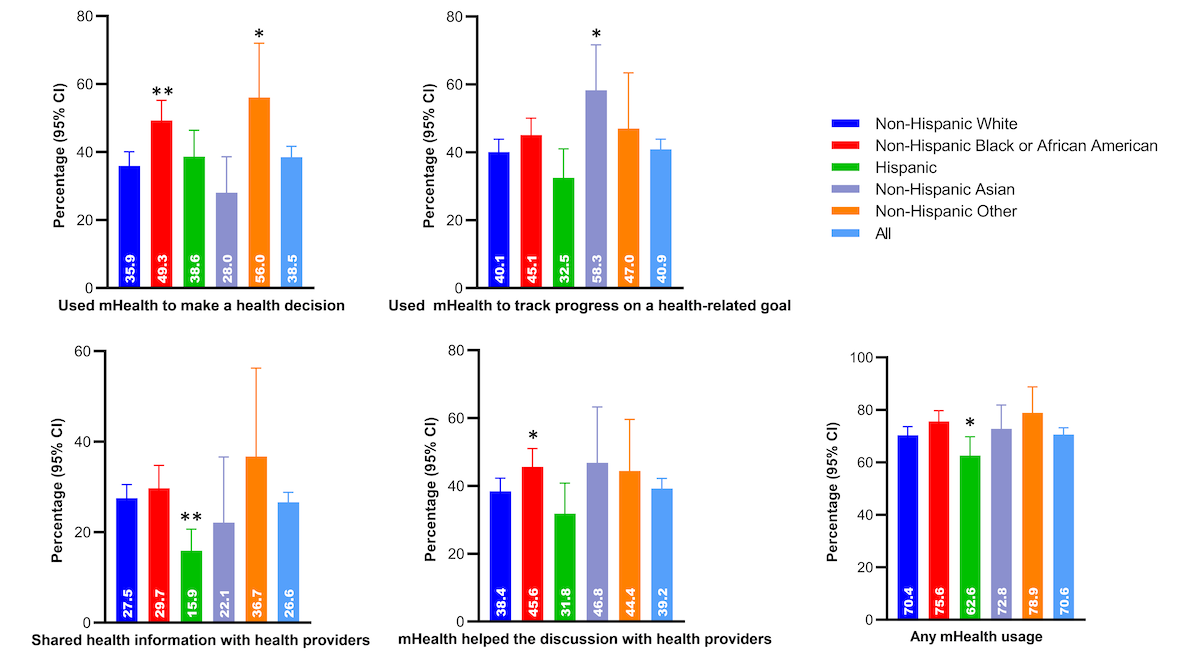
Weighted percentage of mHealth usage among adults with hypertension by race. mHealth: mobile health.

The weighted proportion of non-Hispanic Black adults who used mHealth to make a health decision was higher than in non-Hispanic White adults (49.3% vs 35.9%, *P*<.001) (Figure 1). Non-Hispanic Black adults were also more likely to use mHealth in discussing with health providers (45.6% vs 38.4%, *P*=.04). Non-Hispanic Asian adults were more likely to use mHealth to track progress on a health-related goal (58.3% vs 40.1, *P*=.02) compared to non-Hispanic White adults. Hispanic adults were less likely to use mHealth to share health information with health providers than non-Hispanic White adults (15.9 vs 27.5%, *P*<.001). Table S2 in Multimedia Appendix 1 presents the unweighted percentage of mHealth usage by race and ethnicity.

In the weighted multivariable logistic regression analyses (Table 2), non-Hispanic Black adults were 1.70 (95% CI 1.23-2.34) times more likely to use mHealth to make health decisions, 1.46 (95% CI 1.02-2.08) times more likely to use mHealth to share health information with health providers, 1.38 (95% CI 1.02-1.87) more likely to use mHealth to discuss health decisions with health providers, and 1.62 (95% CI 1.13-2.32) times more likely to use mHealth in any of the four health-related activities, compared to non-Hispanic White adults.

**Table 2:** Associations between race or ethnicity and involvement in shared decision-making or mHealth usage^a^.

Outcome of interest			Non-Hispanic White	Non-Hispanic Black	Hispanic	Non-Hispanic Asian	Non-Hispanic other		
**Always involved in** **SDM^b^ (n=4280)**									
	Unweighted		1.00 (ref)^c^	1.16 (0.96-1.41)	0.90 (0.73-1.12)	*0.58* (*0.41-0.83*)^d^	*0.65* (*0.46-0.91*)		
	Weighted		1.00 (ref)	1.26 (0.89-1.79)	1.06 (0.70-1.59)	*0.51* (*0.26-0.99*)	0.61 (0.33-1.14)		
**mHealth^e^ usage**									
	**Smartphones or tablets helped the discussion with health providers (n=4041)**								
		Unweighted	1.00 (ref)	*1.26* (*1.05-1.51*)	0.89 (0.72-1.11)	1.22 (0.89-1.69)	0.82 (0.58-1.17)		
		Weighted	1.00 (ref)	*1.38* (*1.02-1.87*)	*0.65* (*0.46-0.94*)	1.29 (0.74-2.28)	0.81 (0.41-1.59)		
	**Used smartphones or tablets to make a health decision (n=4040)**								
		Unweighted	1.00 (ref)	*1.40* (*1.17-1.69*)	1.17 (0.95-1.45)	1.18 (0.85-1.63)	1.08 (0.77-1.51)		
		Weighted	1.00 (ref)	*1.70* (*1.23-2.34*)	1.03 (0.73-1.47)	1.01 (0.55-1.87)	1.60 (0.80-3.19)		
	**Used smartphones or tablets to track progress on a health-related goal (n=4047)**								
		Unweighted	1.00 (ref)	*1.49* (*1.23-1.80*)	0.92 (0.73-1.14)	*1.79* (*1.28-2.51*)	1.23 (0.87-1.75)		
		Weighted	1.00 (ref)	1.35 (0.95-1.90)	0.86 (0.58-1.27)	*2.07* (*1.28-3.34*)	1.49 (0.82-2.67)		
	**Shared health information from a smartphone or tablet with health providers (n=4060)**								
		Unweighted	1.00 (ref)	*1.29* (*1.06-1.56*)	*0.68* (*0.53-0.86*)	0.86 (0.60-1.23)	1.04 (0.73-1.50)		
		Weighted	1.00 (ref)	*1.46* (*1.02-2.08*)	*0.47* (*0.33-0.67*)	0.95 (0.53-1.71)	1.36 (0.68-2.75)		
	**Any mHealth usage^f^ (n=4198)**								
		Unweighted	1.00 (ref)	*1.39* (*1.14-1.70*)	0.91 (0.73-1.14)	1.13 (0.78-1.63)	1.14 (0.78-1.66)		
		Weighted	1.00 (ref)	*1.62* (*1.13-2.32*)	0.69 (0.50-0.97)	1.26 (0.73-2.20)	1.67 (0.86-3.22)		

^a^Results from multivariable logistic regression models. Data represent adjusted odds ratio and 95% CI. Each logistic regression model was adjusted for age, sex, education, household income, marital status, location, health insurance, BMI, current smoking status, history of heart condition, diabetes, lung diseases, depression, and cancer.

^b^SDM: shared decision-making.

^c^Ref: reference group.

^d^Italic formatting represents statistical significance.

^e^mHealth: mobile health.

^f^ Any mHealth usage defined as mHealth use in any of the 4 health-related activities described above.

### Association Between Race or Ethnicity and SDM

In the weighted multivariable logistic regression analyses (Table 2), non-Hispanic Asian adults were 0.51 (95% CI 0.26-0.99) times less likely to be involved in SDM. No other significant differences were observed in relation to race or ethnicity and involvement in SDM.

### Association Between Race or Ethnicity and mHealth Usage Among Overall Participants

In the weighted multivariable logistic regression analyses (Table 2), compared to non-Hispanic White adults, non-Hispanic Black adults were found to be 1.38 (95% CI 1.38-1.87) times more likely to use mHealth for discussing health decisions with health providers, 1.70 (95% CI 1.23-2.34) times more likely to make a health decision using mHealth, and 1.62 (95% CI 1.13-2.32) times more likely to engage in any of the 4 health-related activities using mHealth. In addition, non-Hispanic Asian adults were 2.07 (95% CI 1.28-3.34) times more likely to use mHealth to track progress on a health-related goal compared to non-Hispanic White adults. However, compared to non-Hispanic White adults, Hispanic adults were 0.47 (95% CI 0.33-0.67) and 0.65 (95% CI 0.46-0.94) times less likely to use mHealth to share health information and discuss health decisions with health providers, respectively.

### Association Between Race or Ethnicity and mHealth Usage Stratified by SDM

Among adults who were always involved in SDM, non-Hispanic Black adults were 1.90 (95% CI 1.18-3.05) times more likely to use mHealth to make health decisions, 1.61 (95% CI 1.04-2.49) times more likely to share health information with health providers, and 1.88 (95% CI 1.16-3.05) times more likely to use mHealth in any of the 4 health-related activities; and non-Hispanic Asian adults were 3.10 (1.37-7.02) times more likely to use mHealth to track progress on a health-related goal compared to non-Hispanic White adults (Table 3). These associations were insignificant among adults who were usually/sometimes/never involved in SDM. On the contrary, among those who were always involved in SDM, Hispanic adults were 0.55 (95% CI 0.33-0.91) times less likely to use mHealth to share health information with health providers compared to non-Hispanic White adults. Among those who were usually/sometimes/never involved in SDM, Hispanic adults were 0.44 (95% CI 0.24-0.79) times less likely to use mHealth to track progress on a health-related goal, 0.38 (95% CI, 0.18-0.82) times less likely to share health information with health providers, and 0.52 (95% CI 0.31-0.88) times less likely to use mHealth in any of the 4 health-related activities compared to non-Hispanic White adults.

**Table 3:** Associations between race or ethnicity and mHealth usage stratified by SDM^a^.

Outcome of interest			Non-Hispanic White		Non-Hispanic Black				Hispanic					Non-Hispanic Asian					Non-Hispanic other		
			Not always SDM^b,c^		Always SDM^d^		Not always SDM		Always SDM		Not always SDM		Always SDM			Not always SDM		Always SDM			Not always SDM
**Smartphones or tablets helped the discussion with health providers (n=2066)**																					
	Unweighted	1.00 (ref)^e^		1.28 (0.99-2.15)		1.24 (0.91-1.71)		0.96 (0.71-1.30)		0.90 (0.63-1.28)		1.38 (0.81-2.36)			1.44 (0.87-2.38)		0.82 (0.49-1.39)			0.89 (0.54-1.49)	
	Weighted	1.00 (ref)		1.21 (0.79-1.85)		*2.09* (*1.09-3.99*)^f^		0.69 (0.42-1.14)		0.70 (0.37-1.33)		1.39 (0.54-3.57)			1.80 (0.61-5.34)		0.53 (0.19-1.48)			1.05 (0.33-3.35)	
**Used smartphones or tablets to make a health decision (n=2064)**																					
	Unweighted	1.00 (ref)		*1.69* (*1.31-2.19*)		1.02 (0.74-1.40)		1.34 (0.99-1.82)		1.04 (0.74-1.46)		1.41 (0.82-2.41)			1.16 (0.70-1.93)		1.14 (0.68-1.90)			1.06 (0.64-1.74)	
	Weighted	1.00 (ref)		*1.90* (*1.18-3.05*)		1.42 (0.82-2.44)		0.99 (0.64-1.54)		1.06 (0.57-1.95)		0.85 (0.37-1.96)			1.03 (0.35-3.04)		1.45 (0.54-3.90)			1.88 (0.66-5.32)	
**Used smartphone/tablet to track progress on a health-related goal (n=2068)**																					
	Unweighted	1.00 (ref)		*1.65* (*1.27-2.15*)		1.18 (0.85-1.65)		1.02 (0.74-1.40)		*0.62* (*0.43-0.92*)		*2.36* (*1.32-4.22*)			1.51 (0.90-2.54)		1.27 (0.74-2.18)			1.30 (0.77-2.19)	
	Weighted	1.00 (ref)		1.37 (0.87-2.14)		0.98 (0.49-1.94)		0.89 (0.50-1.58)		*0.44* (*0.24-0.79*)		*3.10* (*1.37-7.02*)			1.42 (0.60-3.38)		2.01 (0.78-5.21)			1.47 (0.64-3.34)	
**Shared health information from a smartphone or tablet with health providers (n=2104)**																					
	Unweighted	1.00 (ref)		*1.34* (*1.04-1.73*)		1.26 (0.90-1.76)		*0.71* (*0.51-0.98*)		*0.61* (*0.40-0.93*)		0.96 (0.55-1.68)			0.99 (0.57-1.70)		0.93 (0.53-1.61)			1.13 (0.66-1.94)	
	Weighted	1.00 (ref)		*1.61* (*1.04-2.49*)		1.14 (0.58-2.25)		*0.55* (*0.33-0.91*)		*0.38* (*0.18-0.82*)		0.59 (0.23-1.52)			1.22 (0.54-2.75)		1.42 (0.45-4.50)			1.27 (0.40-3.99)	
**Any mHealth^g,h^ usage (n=2160)**																					
	Unweighted	1.00 (ref)		*1.48* (*1.10-1.96*)		1.16 (0.82-1.64)		0.95 (0.68-1.31)		0.75 (0.52-1.07)		1.30 (0.66-2.54)			1.21 (0.68-2.15)		1.30 (0.70-2.42)			1.10 (0.64-1.89)	
	Weighted	1.00 (ref)		*1.88* (*1.16-3.05*)		1.13 (0.56-2.29)		0.68 (0.40-1.18)		*0.52* (*0.31-0.88*)		1.02 (0.38-2.73)			1.37 (0.53-3.55)		*3.12* (*1.23-7.93*)			1.41 (0.52-3.83)	

^a^Results from multivariable logistic regression models. Data represent adjusted odds ratio and 95% CI. Each logistic regression model was adjusted for age, sex, education, household income, marital status, location, health insurance, BMI, current smoking status, history of heart condition, diabetes, lung diseases, depression, and cancer.

^b^Not always SDM: among people who were usually/sometimes/never involved in SDM (n=1768).

^c^SDM: shared decision-making.

^d^Always SDM: among people who were always involved in SDM (n=2512).

^e^Ref: reference group.

^f^Italic formatting represents statistical significance.

^g^Any mHealth usage is defined as mHealth use in any of the 4 health-related activities described above.

^h^mHealth: mobile health.

### Association Between mHealth Usage and SDM

Adults who used mHealth to track progress on a health-related goal were 1.35 (95% CI 1.03-1.78) times more likely always to be involved in SDM than those who did not (Table 4). Using mHealth to share health information with health providers was marginally associated with SDM 1.23 (95% CI 0.98-1.55).

**Table 4:** Associations between mobile health (mHealth) usage and shared decision-making^a^.

mHealth usage		Unweighted aOR^b^ (95% CI)	Weighted aOR (95% CI)
**Model 1: smartphones or tablets helped the discussion with health providers (n=3648** **)**			
	No	1 (ref)^c^	1 (ref)
	Yes	1.13 (0.98-1.30)	1.14 (0.90-1.45)
**Mode 2: used smartphone or tablet to make a health decision (n=3652)**			
	No	1 (ref)	1 (ref)
	Yes	1.02 (0.89-1.17)	1.06 (0.85-1.32)
**Model 3: used smartphone or tablet to track progress on a health-related goal (n=3660)**			
	No	1 (ref)	1 (ref)
	Yes	*1.22* (*1.06-1.41*)^d^	*1.35* (*1.03-1.78*)
**Model 4: shared health information from a smartphone/tablet with health providers (n=4142)**			
	No	1 (ref)	1 (ref)
	Yes	*1.27* (*1.10-1.46*)	1.23 (0.98-1.55)
**Model 5: any mHealth^e^ usage (n=3688)**			
	No	1 (ref)	1 (ref)
	Yes	1.07 (0.92-1.25)	1.06 (0.86-1.31)

^a^Results from multivariable logistic regression models. The outcome of interest of each model is always involved in shared decision-making. The exposure of interest of each model is mHealth usage. Each logistic regression model is adjusted for age, sex, education, household income, marital status, location, health insurance, BMI, current smoking status, history of heart condition, diabetes, lung diseases, depression, and cancer.

^b^aOR: adjusted odds ratio.

^c^Ref: reference group.

^d^Italics formatting represents statistical significance.

^e^mHealth: mobile health.

## Discussion

### Principal Results

In this cross-sectional study of US adults with self-reported hypertension, we found racial or ethnic disparities in SDM and mHealth usage. Non-Hispanic Asian adults were less likely to be involved in SDM compared with non-Hispanic White adults. Non-Hispanic Black adults, particularly those who were always involved in SDM, were more likely to use mHealth to make a health decision and discuss health decisions with health providers compared with non-Hispanic White adults. Non-Hispanic Asian adults, especially those who were always involved in SDM, were more likely to use mHealth to track progress on a health-related goal than non-Hispanic White adults. Hispanic adults were less likely to use mHealth to share health information with health providers, regardless of their level of involvement in SDM.

### Comparison With Prior Work

Hypertension control rates in the United States have declined over the past decade, with significantly lower rates of control among people from racial and ethnic minority groups [2,5]. SDM has been acknowledged as a valuable approach to enhancing hypertension control by promoting patient involvement in health care decisions and facilitating patient-centered care [3]. Additionally, SDM has shown the potential to reduce inequalities in hypertension management by improving patient-provider communication, particularly among minority populations [6,11]. Previous research has consistently shown that minority populations, such as Black individuals, tend to experience lower communication quality, receive less information, have limited patient participation, and engage in less participatory SDM compared to White patients [8]. However, our study did not observe a significant difference in SDM involvement between Black and White adults. Interestingly, our findings revealed that Asian adults were less likely to be involved in SDM compared to White adults. It is important to note that while the single-item measure of individuals’ engagement in patient-provider discussion used in our study provides valuable insights into the prevalence of participant involvement in health care decisions, it does not capture the full complexity of SDM [41]. Thus, it should be considered a proxy measure. To achieve a thorough understanding of this topic, further research is necessary to use measures that specifically assess SDM within the context of discussions pertaining to hypertension control among adults from various racial and ethnic backgrounds.

The advantages of mHealth technology, such as ease of access, real-time feedback, and feasibility, have facilitated its usage in improving hypertension control [42,43]. Moreover, the feasibility of mHealth solutions makes them accessible to a broader population, irrespective of geographic location, or socioeconomic status [21]. Implementing mHealth technology in populations with disparities in digital health use presents an opportunity to address health disparities in hypertension management [21]. Several studies investigating mHealth usage among older adults have found significant disparities, with minorities being less likely than non-Hispanic Whites to own mHealth devices (eg, computers and smartphones), use the internet and email, and have the ability and willingness to engage in health care–related activities using mHealth technology [28,29]. Our study, for the first time, focused on the use of mHealth technology in supporting 4 health-related activities among adults with hypertension. Our study yielded unexpected findings, revealing higher mHealth usage among Black and Asian adults with hypertension, especially among those who were always involved in SDM, compared to White adults. This apparent contradiction raises significant questions regarding the effectiveness of mHealth technology in addressing the specific barriers faced by marginalized ethnic groups when it comes to hypertension control [21,29].

While SDM interventions and mHealth usage may have a positive impact on hypertension control overall, they might not adequately address the unique challenges encountered by marginalized ethnic groups, resulting in persistent disparities in hypertension control [6]. Indeed, multiple factors contribute to the existence of racial and ethnic disparities in hypertension control, including social determinants of health (limited access to health care, low health literacy, lower socioeconomic status, etc), clinical inertia (eg, lower treatment rates), and biological factors (salt sensitivity) [6]. When considering the effectiveness of mHealth technology–based interventions in addressing health disparities, it is essential to acknowledge that these interventions have the potential to exacerbate existing disparities. This is particularly true for historically minoritized populations, as they may face decreased access to the internet, which is a critical prerequisite for using mHealth interventions effectively. However, research has shown that certain racial and ethnic minority groups, as well as individuals from low-income backgrounds, may experience limited access to the internet compared to White adults [44-46]. This may result in the “digital divide” causing populations that have poorer health outcomes to continue having poorer health outcomes, despite available technological improvements [47,48]. A previous study revealed that Asians and Black adults were less likely than White people to access the internet using a personal computer [49]. A recent study using US national household data from the American Community Survey and the Current Population Survey showed that low-income non-Hispanic Black and Hispanic youth were the most likely to lack home internet access [50]. Additionally, acculturation, language, and immigration status can result in various barriers to health care access in the Hispanic and many immigrant populations [51]. For instance, a high prevalence of low health literacy is frequently found among Spanish-speaking adults [52]. In addition, an English-dominant health care system may impose further barriers on Hispanic individuals with low English proficiency and limited health literacy [53,54]. In a study on the availability of Spanish-language medical apps, only 10% of apps were in Spanish and met the inclusion criteria [55]. These are important issues, given the connection between low health literacy and the exacerbation of health disparities [56].

Therefore, while SDM and mHealth interventions have shown potential, it is essential to consider and address the broader determinants of hypertension control to effectively tackle disparities experienced by marginalized ethnic groups [57]. As advances are achieved in the use of mHealth technology, it is important to intentionally create strategies and features that promote the inclusion of all populations to avoid the digital divide and equitably promote improvements in health outcomes for all [29]. To effectively reduce inequities in hypertension management through mHealth technology and SDM interventions (especially carried out via mHealth tools), it is crucial to address any racially distributed barriers that may hinder optimal blood pressure control, such as social determinants of health [6,21]. Additionally, for an intervention to truly mitigate disparities, it must demonstrate greater efficacy among marginalized groups compared to advantaged groups [29].

### Strengths and Limitations

This study is subject to limitations. First, this analysis is subject to a limitation due to the use of a single item to measure SDM. The question focused on concordance between desired and received involvement, rather than directly assessing active engagement in decision-making. This measurement approach may not fully capture the complexity and nuances of SDM. Furthermore, our analysis did not account for potential ethnic and racial differences in response patterns and preferences for involvement in SDM. Additionally, factors such as cultural safety, which influence individuals' comfort and trust in the health care setting, were not explicitly considered in our analysis. Second, HINTS is a cross-sectional study, and thus, temporal associations cannot be established. In addition, because this is a secondary data analysis, we lacked information on other potential confounders, such as health literacy (2 items that might be considered to determine health literacy [36] were only available in 2017 and 2019). Third, HINTS outcomes are based on self-reported diagnoses of hypertension. Therefore, participants’ understanding of their health condition could have affected reported data, in addition to the issues of lack of health care access and social desirability, which could result in an underestimation of the true prevalence.

Despite the limitations, our study presents remarkable strengths. This study evaluates a large nationally representative sample of noninstitutionalized civilians. In addition, this study used 4 years of pooled data to increase statistical power and provide meaningful comparisons across race or ethnic groups. HINTS enables the participation of Spanish-speaking Latino individuals by administering the survey in English and Spanish. This population-based study also addresses a gap in health research, providing novel insights into the effects of racial or ethnic disparities in the usage of mHealth technology or SDM usage among adults with hypertension.

### Conclusions

Our study of US adults with hypertension found that Asian adults exhibited lower engagement in SDM compared to their White counterparts. Additionally, we observed higher usage of mHealth technology among Black and Asian adults compared to White adults. These findings emphasize the significance of comprehending the involvement of SDM and usage of mHealth technology within racially and ethnically diverse populations. Such understanding is essential for the development of targeted interventions aimed at improving minority health and addressing health disparities. However, there is a need for further research comparing racial disparities in the usage of mHealth technology to support SDM in hypertension management.

## Appendix

Multimedia Appendix 1 Unweighted demographic characteristics and clinical data among adults with hypertension and unweighted number and percentage of mHealth usage among adults with hypertension by race and ethnicity.

## Abbreviations

aOR: adjusted odds ratio
HC: health communication
HINTS: Health Information National Trends Survey
HIT: health information technology
mHealth: mobile health
SDM: shared decision-making
