# Supplementary material for: Racial Disparities in Shared Decision-Making and the Use of mHealth Technology Among Adults With Hypertension in the 2017-2020 Health Information National Trends Survey: Cross-Sectional Study in the United States
Source: J Med Internet Res. 2023 Sep 13;25:e47566. doi: 10.2196/47566 (PMC10534288; doi:10.2196/47566)
Supplement: Multimedia Appendix 1 [file jmir_v25i1e47566_app1.docx]

**Table S1.** Unweighted demographic characteristics and clinical data among adults with hypertension.

| **Characteristics** | | **All (N=4893)**  **n (%)** | **Non-Hispanic White (n=3006)**  **n (%)** | **Non-Hispanic Black (n=907)**  **n (%)** | **Hispanic (n=605)**  **n (%)** | **Non-Hispanic Asian (n=193)**  **n (%)** | **Non-Hispanic Other (n=182)**  **n (%)** | ***P*** |
| --- | --- | --- | --- | --- | --- | --- | --- | --- |
| Age, Mean (SD) | | 61.3 (13.2) | 62.9 (12.9) | 59.1 (12.6) | 57.8 (13.7) | 60.0 (14.4) | 57.1 (13.3) | < 0.001 |
| Age | | | | | |  |  | < 0.001 |
|  | 18-34 years | 195 (4.0) | 96 (3.2) | 42 (4.8) | 32 (5.4) | 12 (6.3) | 13 (7.3) |  |
|  | 35-49 years | 643 (13.3) | 318 (10.7) | 140 (15.8) | 123 (20.6) | 34 (17.8) | 28 (15.7) |  |
|  | 50-64 years | 1861 (38.6) | 1088 (36.6) | 384 (43.4) | 242 (40.6) | 62 (32.5) | 85 (47.8) |  |
|  | 65-74 years | 1416 (29.4) | 947 (31.8) | 242 (27.4) | 140 (23.5) | 50 (26.2) | 37 (20.8) |  |
|  | 75+ years | 708 (14.7) | 525 (17.7) | 76 (8.6) | 59 (9.9) | 33 (17.3) | 15 (8.4) |  |
| Sex | | | | | |  |  | < 0.001 |
|  | Female | 2491 (53.2) | 1456 (50.5) | 574 (67.0) | 274 (47.5) | 80 (41.9) | 107 (61.9) |  |
|  | Male | 2189 (46.8) | 1426 (49.5) | 283 (33.0) | 303 (52.5) | 111 (58.1) | 66 (38.2) |  |
| Education | | | | | |  |  | < 0.001 |
|  | Less than high school | 264 (5.4) | 91 (3.0) | 52 (5.8) | 95 (15.9) | 11 (5.7) | 15 (8.2) |  |
|  | High school graduate | 862 (17.7) | 524 (17.5) | 193 (21.5) | 111 (18.5) | 11 (5.7) | 23 (12.6) |  |
|  | Some college | 1664 (34.2) | 1027 (34.4) | 322 (35.9) | 214 (35.7) | 30 (15.6) | 71 (39.0) |  |
|  | Bachelor's degree | 2071 (42.6) | 1348 (45.1) | 179 (29.9) | 179 (29.9) | 140 (72.9) | 73 (40.1) |  |
| Household income | | | | | |  |  | < 0.001 |
|  | < $20 000 | 760 (16.8) | 348 (12.6) | 230 (27.5) | 115 (20.3) | 20 (10.6) | 47 (26.9) |  |
|  | $20 000-$35 000 | 624 (13.8) | 336 (12.2) | 154 (18.4) | 99 (17.4) | 19 (10.1) | 16 (9.1) |  |
|  | $35 000-$50 000 | 652 (14.4) | 373 (13.5) | 124 (14.9) | 98 (17.3) | 27 (14.4) | 30 (17.1) |  |
|  | $50 000-$75 000 | 885 (19.5) | 572 (20.7) | 150 (18.0) | 93 (16.4) | 37 (19.7) | 33 (18.9) |  |
|  | ≥ $75 000 | 1608 (35.5) | 1134 (41.0) | 177 (21.2) | 163 (28.7) | 85 (45.2) | 49 (28.0) |  |
| Marital Status | | | | | |  |  | < 0.001 |
|  | Married | 2311 (47.6) | 1299 (43.5) | 578 (64.5) | 279 (46.8) | 48 (25.0) | 107 (59.1) |  |
|  | Others^&^ | 2541 (52.4) | 1688 (56.5) | 318 (35.5) | 317 (53.2) | 144 (75.0) | 74 (40.9) |  |
| Insurance | | | | | |  |  | < 0.001 |
|  | No | 164 (3.4) | 65 (2.2) | 45 (5.0) | 42 (7.0) | 4 (2.1) | 8 (4.4) |  |
|  | Yes | 4686 (96.6) | 2919 (97.8) | 853 (95.0) | 556 (93.0) | 185 (97.9) | 173 (95.6) |  |
| Location | | | | | |  |  | < 0.001 |
|  | Urban | 4284 (87.6) | 2535 (84.3) | 837 (92.3) | 563 (93.1) | 187 (96.9) | 162 (89.0) |  |
|  | Rural | 609 (12.4) | 471 (15.7) | 70 (7.7) | 42 (6.9) | 6 (3.1) | 20 (11.0) |  |
| BMI | | | | | | | | < 0.001 |
|  | < 25 kg/m^2^ | 922 (18.8) | 616 (20.5) | 118 (13.0) | 79 (13.1) | 81 (42.0) | 28 (15.4) |  |
|  | ≥ 25 kg/m^2^ | 3971 (81.2) | 2390 (79.5) | 789 (87.0) | 526 (86.9) | 112 (58.0) | 154 (84.6) |  |
| Current smoking | | | | | | | | < 0.001 |
|  | No | 4209 (86.6) | 2587 (86.6) | 784 (87.5) | 525 (87.4) | 172 (89.1) | 141 (77.5) |  |
|  | Yes | 652 (13.4) | 402 (13.4) | 112 (12.5) | 76 (12.6) | 21 (10.9) | 41 (22.5) |  |
| Depression | | | | | | | | < 0.001 |
|  | No | 3617 (74.2) | 2193 (73.3) | 709 (78.7) | 438 (72.6) | 169 (87.6) | 108 (59.7) |  |
|  | Yes | 1255 (25.8) | 801 (26.8) | 192 (21.3) | 165 (27.4) | 24 (12.4) | 73 (40.3) |  |
| Heart condition | | | | | | | | 0.072 |
|  | No | 4108 (84.2) | 2490 (83.0) | 782 (86.5) | 517 (85.9) | 166 (86.0) | 153 (84.5) |  |
|  | Yes | 772 (15.8) | 510 (17.0) | 122 (13.5) | 85 (14.1) | 27 (14.0) | 28 (15.5) |  |
| Diabetes | | | | | | | | < 0.001 |
|  | No | 3148 (65.0) | 2056 (69.0) | 540 (60.6) | 325 (54.0) | 107 (56.3) | 120 (66.7) |  |
|  | Yes | 1696 (35.0) | 925 (31.0) | 351 (39.4) | 277 (46.0) | 83 (43.7) | 60 (33.3) |  |
| Lung disease | | | | | | | | 0.001 |
|  | No | 4113 (84.3) | 2542 (84.7) | 761 (84.4) | 508 (87.6) | 169 (87.6) | 133 (73.5) |  |
|  | Yes | 768 (15.7) | 459 (15.3) | 141 (15.6) | 24 (12.4) | 24 (12.4) | 48 (26.5) |  |
| Cancer | | | | | | | | < 0.001 |
|  | No | 3919 (80.4) | 2278 (76.0) | 778 (86.4) | 536 (88.9) | 174 (90.6) | 153 (84.1) |  |
|  | Yes | 954 (19.6) | 718 (24.0) | 122 (13.6) | 67 (11.1) | 18 (9.4) | 29 (15.9) |  |
| SDM | | | | | | | | < 0.001 |
|  | Usually/Sometimes/Never | 1768 (41.3) | 1098 (40.9) | 281 (35.8) | 222 (44.5) | 81 (55.1) | 86 (52.4) |  |
|  | Always | 2512 (58.7) | 1587 (58.1) | 504 (64.2) | 277 (55.5) | 66 (44.9) | 78 (47.6) |  |
| BMI: body mass index. CVD: cardiovascular diseases. SD: standard deviation. SDM, shared decision-making. ^&^Including divorced, widowed, separated, living as married, and single (never been married). | | | | | | | | |

**Table S2.** Unweighted number and percentage of mHealth usage among adults with hypertension by race and ethnicity.

|  | **All** | **Non-Hispanic White** | **Non-Hispanic Black** | **Hispanic** | **Non-Hispanic Asian** | **Non-Hispanic Other** | ***P*** |
| --- | --- | --- | --- | --- | --- | --- | --- |
| **Used smartphone/tablet to make a health decision** | | | | | | | < 0.001 |
| No | 2905 (61.7) | 1876 (64.7) | 480 (55.7) | 339 (58.4) | 108 (57.5) | 102 (57.6) |  |
| Yes | 1804 (38.3) | 1025 (35.3) | 382 (44.3) | 242 (41.7) | 80 (42.6) | 75 (42.4) |  |
| **Used smartphone/tablet to track progress on a health-related goal** | | | | | | | 0.001 |
| No | 2950 (62.6) | 1893 (65.2) | 488 (56.5) | 376 (64.8) | 89 (47.3) | 104 (58.8) |  |
| Yes | 1764 (37.4) | 1012 (34.8) | 376 (43.5) | 204 (35.2) | 99 (52.7) | 73 (41.2) |  |
| **Shared health information from a smartphone/tablet with health providers** | | | | | |  | 0.001 |
| No | 3319 (70.3) | 2034 (69.8) | 589 (67.4) | 444 (77.5) | 134 (72.0) | 118 (68.6) |  |
| Yes | 1400 (29.7) | 880 (30.2) | 285 (32.6) | 129 (22.5) | 52 (28.0) | 54 (31.4) |  |
| **Smartphones/tablets helped the discussion with health providers** | | | | | | | 0.007 |
| No | 2876 (61.1) | 1796 (61.9) | 498 (57.8) | 374 (64.4) | 98 (52.1) | 110 (62.5) |  |
| Yes | 1832 (38.9) | 1105 (38.1) | 364 (42.2) | 207 (35.6) | 90 (47.9) | 66 (37.5) |  |
| **Any mHealth usage**^‡‡^ | | | | | | | 0.006 |
| No | 1479 (30.2) | 951 (31.6) | 236 (26.0) | 193 (31.9) | 48 (24.9) | 51 (28.0) |  |
| Yes | 3414 (69.8) | 2055 (68.4) | 671 (74.0) | 412 (68.1) | 145 (75.1) | 131 (72.0) |  |

^‡‡^ Any mHealth usage defined as mHealth use in any of the four health-related activities described above.

mHealth: mobile health.
